# UV-Visible spectroscopic study on multi-staged film formation mechanisms of graphene oxide-doped polystyrene latex (PS latex/GO) nanocomposites

**DOI:** 10.3906/kim-2102-30

**Published:** 2021-03-31

**Authors:** Asef ETEMADI, Barış DEMİRBAY, Şaziye UĞUR

**Affiliations:** Department of Physics Engineering, Faculty of Science and Letters, İstanbul Technical University, İstanbul, Turkey

**Keywords:** Void closure, interdiffusion, polystyrene latex, graphene oxide, polymer nanocomposites, polymer physics

## Abstract

We report the effect of graphene oxide (GO) on film formation and morphological properties of GO-doped polystyrene (PS) latex nanocomposite (PS latex/GO) films using UV-visible absorption spectroscopy and scanning electron microscopy (SEM). PS latex particles were synthesized through emulsion polymerization technique and then nanocomposite blends, each containing different concentrations of GO ranging from 0 wt% to 70 wt%, were obtained. Prepared blends were deposited on glass plates via drop-casting method, and coated substrates were annealed at different temperatures between 100 °C and 250 °C. At each annealing temperature, transmitted light intensity of nanocomposite films was recorded. Void closure and Prager–Tirrell models were employed to interpret the film formation behavior. Activation energy of viscous flow (Δ*H*) decreased from 21.80 kcal·mol^−1^ to 5.91 kcal·mol^−1^ when the amount of GO content increased in film composition. However, activation energy of interdiffusion (Δ*E*) varied between 1.08 kcal·mol^−1^ and 6.94 kcal·mol^−1^ without any trend upon the addition of GO nanofillers. SEM images of films agreed well with calculated activation energies. Although the interdiffusion process of GO-doped latex films remained unaffected by added GO nanofillers, optical transparency of the films enhanced up to 92.5%, demonstrating that thermally resistant and highly transparent GO-rich nanocomposite films can be fabricated.

## 1. Introduction

Polymer latexes, also referred to as colloidal polymer particles uniformly dispersed in a carrier medium, are of utmost importance to researchers for many decades due to their high performance and remarkable features. Such colloidal latexes have a wide array of applications in the engineering industry and are most commonly utilized as a binder and film-forming agents in composite materials [1–3] since they exhibit excellent optical properties even in the presence of reinforcing nanofillers [4]. In this regard, the film formation of latex-based coating compounds is a significant feature that needs to be considered in all latex and composite applications. Latex film formation is a complex and multi-stage physical phenomenon that shows a difference based upon the structure of latex particles. Film formation mechanisms of latex particles, which have a low glass transition temperature (*T_g_*) referring to soft latex particles, and high-*T_g_* (hard) latex particles, can take place in two main stages [5]. For both types of latex particles, the wet initial stage is known as the first stage. Then, solvent evaporation gives rise to the second film formation stage where the latex particles form a close-packed array. In this stage, the soft latex particles are deformed to polyhedrons whereas the hard latex particles keep their structure the same without deforming. The annealing process of soft latex particles leads to diffusion across particle-particle boundaries resulting in a homogeneous material. As distinct from soft latex particles, during the annealing of high-*T_g_* latexes, particle deformation results in, first, void closure phenomenon [6,7], and then the voids begin to disappear while the diffusion process across particle-particle boundaries emerges. Upon the sample annealing, the mechanical properties of high-*T_g_* latex films can evolve due to the evaporation of all solvent and void closure is completed.

The addition of nanofillers into polymer latexes can remarkably improve their mechanical, thermal and electrical properties, and endow multifunctionality together with other superior features to the polymer matrix. Such reinforced, multi-phased composite materials where at least one of the phases must be in the range between 1–50 nm, are known as polymer nanocomposites. Polymer nanocomposites can exhibit substantial characteristic enhancements even in the presence of pretty low nanofiller loadings in comparison with standard polymer composites containing micron-scale fillers, such as glass or carbon fibers [8]. In recent years, graphene, the 2-D honeycomb lattice composed of sp^2^-bonded carbons, has garnered considerable scientific attention due to its novel molecular structure, excellent physical, and electronic characteristics [9]. Yet, as the graphene nanolayers strongly prone to form agglomerations, this can turn out to be a major problem in its practical applications. In comparison with other forms of graphene-based materials, graphene oxide (GO) is a great candidate to be utilized as the reinforcing filler within polymer matrices since GO has a low cost, unique molecular structure, and can be easily fabricated [10,11]. Furthermore, since GO contains multiple oxygen moieties on its molecular structure, it has greater characteristics than graphene such as high hydrophilicity and tunable electronic features. On top of that, in order to fabricate high-performance carbon-based nanomaterials, the molecular structure of GO can be further functionalized by employing specific chemical compounds. Taking into account these remarkable properties, GO can thus be considered as a very promising nanofiller for the fabrication of the most advanced multifunctional composite materials. However, there are various limiting aspects obstructing the efficiency of GO nanolayer-doped nanocomposites. Recent GO-related studies have revealed that GO nanolayers have a strong tendency to stacking, folding or buckling when they are utilized as nanofillers within a polymeric medium [12]. Moreover, the proper selection of the fabrication and synthesis methods can also strongly affect the physical characteristics together with the surface morphology of GO-reinforced nanocomposite materials. For this reason, one needs to ensure a homogeneous GO layer dispersion to be able to enhance the performance of GO in polymer nanocomposites.

Solution mixing is simple and one of the widely used methods addressed for the preparation of GO-doped nanocomposites. It is an effective method to make sure very uniform GO dispersions, however, it is also known that this method is not quite environmentally friendly and may not always compatible with production on large-scale. To circumvent such problems, a novel method based on latex technology using a solvent-free approach has recently been developed for the synthesis of carbon-based composite materials with remarkable properties including carbon nanotubes (CNTs) [13] and carbon black (CB) [14]. Such an effective latex technology is not limited only for CNT or CB-doped composites; however, it has also been used for the preparation of various polymer nanocomposites reinforced with graphene and its derivatives. It is known that graphene-doped polymer nanocomposites with high efficiency can be easily synthesized through mixing the polymers together with well-dispersed graphene suspensions [15,16]. For such a significant nanocomposite design with high throughput efficiency, the latex technology enables the incorporation of homogeneously dispersed GO nanolayers within high viscous polymer matrices, for example, poly(methyl methacrylate) (PMMA) and polystyrene (PS). Up to date, several different high-performance GO-doped polymer nanocomposites could be successfully fabricated, characterized, and reported in the academic literature by many researchers. Zhong et al. have synthesized PS/GO composites and could manage to enhance the storage modulus as well as the thermal stability of neat PS itself by the addition of GO nanolayers into PS environment [17]. Yang and co-workers could manage to prepare novel layer-structured poly(vinyl alcohol) (PVA)/GO composites and they could remarkably enhance both mechanical and thermal characteristics of PVA in the presence of GO nanolayers [18]. In addition to these scientific contributions, Shi et al. have carried out a novel solvent exchange technique for the fabrication of polybenzimidazole/GO composites, and they could obtain a full exfoliation of GO nanofillers even at very high concentrations in the presence of an organic solvent during fabrication process [19]. All these novel, significant contributions reported by researchers commonly point out that the characteristics of the used polymer itself can be greatly improved if one can properly ensure a uniform, well-dispersed GO nanolayers within a polymeric medium. For this reason, it is required to make sure uniform GO nanofiller dispersions embedded within the polymer materials, and one has to aware of the interfacial binding.

In the present research, the influence of the GO nanofillers on film formation mechanisms, each taking place at different annealing temperatures, and morphological properties of the nanocomposites was studied by using UV-visible spectroscopy and scanning electron microscopy (SEM) modalities. Before performing the measurements, emulsion mixing and drop-casting methods were used as nanocomposite preparation methods owing to their simplicity and versatility. As colloidal PS latex is a widely used multifunctional and thermally stable industrial polymer even at very high annealing temperatures, it was employed as a matrix phase together with GO nanofillers at different concentrations in nanocomposite films. Experimental information including chemical compounds, sample preparation as well as measurement results were provided with further details in the following sections.

## 2. Experimental procedures

### 2.1. Materials

**Figure 1:**
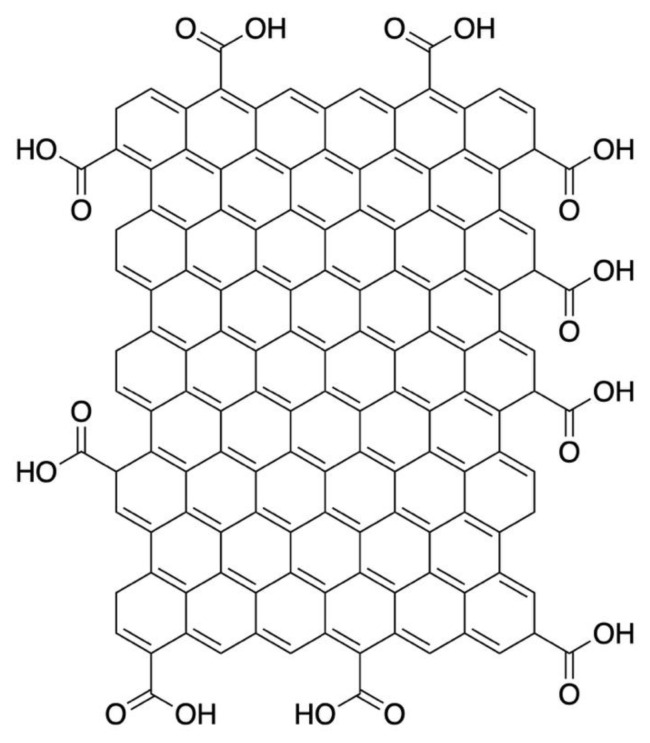
The chemical structure of graphene oxide (GO).

**Figure 2:**
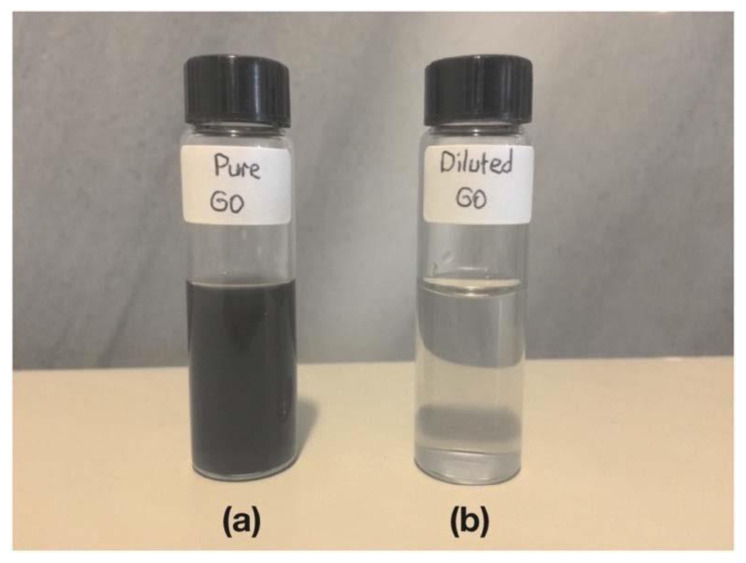
Graphene oxide (GO) dispersions: (a) stock solution (left) and (b) after dilution and ultrasonication process (right).

Graphene oxide (GO) stock solution (purchased from Sigma-Aldrich, Product No: 794341) (Sigma-Aldrich Corp., St. Louis, MO, USA) was used as aqueous dispersions at 1 mg/mL and its chemical structure is given in Figure 1. In the present research, the GO solution was used directly without any purification process. In order to prepare aqueous GO suspensions at 0.5 mg/mL through a sonication process, the original GO stock suspension (dark brown-colored as shown in Figure 2a) was first redispersed in distilled (dH_2_O) water. After that sonicated GO solution was mechanically stirred for about 10 min at room temperature. Figure 2a and Figure 2b show the effect of the sonication process on GO dispersions before and after the dilution. As one can apparently see from Figure 2b that GO was exfoliated in the water where neither sedimentation nor aggregation was observed. The transparent, well-exfoliated GO dispersion (see Figure 2b) was successfully obtained as a result of a gentle sonication process together with mechanical stirring. The PS latex particles employed in this research were synthesized by an emulsion polymerization method [20].

The polymerization technique was batch-wisely carried out by the use of a thermo-stated reactor which is attached to a condenser, mechanical stirring paddle, thermocouple and a nitrogen inlet. As the first step, 0.014 g of fluorescent 1-pyrenylmethyl methacrylate (PolyFluor 394), 3 g of styrene monomer and 50 ml of water were mixed within the polymerization reactor at constant temperature of 70 °C. In order to induce the styrene polymerization, as a radical initiator, potassium persulfate (1.6% wt/wt over styrene) which was dissolved in 2 mL of water, was added into reactor. After that, as a surfactant ingredient, 0.075% (wt/vol) of sodium dodecyl sulfate was then introduced in polymerization recipe. The entire polymerization process was governed under 400 rpm agitation for about 12 h in the nitrogen environment at the same temperature (70 °C). Malvern Instrument NanoZS was used to determine the particle diameter of the synthesized PS latex particles. As a result, the mean diameter of PS latex particles was found to be around 540 nm. After that gel permeation chromatography was used to measure the weight-average molecular weight (*M_w_*) of individual PS chain and *M_w_* was found to be 3.32 × 10^5^ g/mol. As the last step, a differential scanning calorimeter was used to determine the glass transition temperature (*T_g_*) of the PS latex particles and it was obtained as about 105 °C which agreed very well with the measured *T_g_* values of PS polymer with and without GO content reported in other experimental studies. Yang et. al have found that *T_g_* value of PS does not exceed 105.9 °C when GO nanofillers are added at high concentrations [21].

In this regard, we also expected the similar *T_g_* values nearly around 105°C for GO-doped nanocomposite latex films that we prepared and the sample annealing was therefore carried out above that of measured *T_g_* value.

### 2.2. Nanocomposite film preparation

A homogeneous PS latex solution at 0.5 mg/mL was prepared on a magnetic stirrer and mixed for 2 h. Afterward, pre-processed sonicated GO solution at a concentration above 0.5 mg/mL was mixed together with PS latex solution on a magnetic stirrer for about 1 h in order to prepare various PS latex/GO suspensions, each containing different mass fractions of GO in the range between 0 wt% and 70 wt%. Each prepared PS latex/GO suspension mixture was first sonicated for 10 min at room temperature and then mechanically stirred at least for 1 h to ensure homogenous mixtures. Transparent glass slides, which were truncated into the dimensions of 0.8 × 2.5 cm^2^, were employed as substrates to be coated by homogeneous PS latex/GO nanocomposite mixtures.

Each surface of the reference glass substrates was gently cleaned using the acetone solution and washed with deionized water several times. After that, all cleansed glass substrates were placed in an oven and dried for 10 min. The dried glass substrates were then coated by using homogeneous PS latex/GO mixtures via drop-casting method and all coated substrates were kept on the workbench to dry completely at room temperature for 12 h. The next day, each dried nanocomposite film substrate was weighed and compared with the weight of empty (uncoated) glass substrates to calculate the film thickness values. The average nanocomposite film density as well as the film thickness of each prepared nanocomposite film can be found from Table 1. In order to investigate the film formation mechanisms of PS latex/GO nanocomposites in detail, each prepared film sample was annealed above *T_g_* for about 10 min one by one at various annealing temperatures in the range between 100 °C and 250 °C. Later on, all nanocomposite substrates were cooled down to room temperature where the temperature in an oven could be maintained within ± 2 °C during the annealing process.

### 2.3.Instrumentation

Photon transmission measurements were performed through using UV–Visible (UVV) scanning spectrophotometer (from Varian Cary 100). The optical transmittance, i.e. transmitted light intensity, *I_tr_*, values of the annealed nanocomposite films were recorded at 500 nm in which neither PS latex nor GO have specific absorption at this wavelength. For all UVV experiments, the truncated glass plates were employed as a standard and optical transmittance of each glass plate that is coated with prepared nanocomposite droplets composed of different mass fractions of GO was then sequentially recorded in spectrometer directly after sample annealing at each temperature. In addition to UVV experiments, in order to monitor the film formation processes, the surface morphology of PS latex/GO nanocomposite films annealed at different temperatures was investigated with further details by performing scanning electron microscopy (SEM) (EVO LS 10 from Zeiss) imaging.

## 3. Results and discussion

### 3.1. Film formation behavior of PS latex/GO nanocomposites

**Figure 3:**
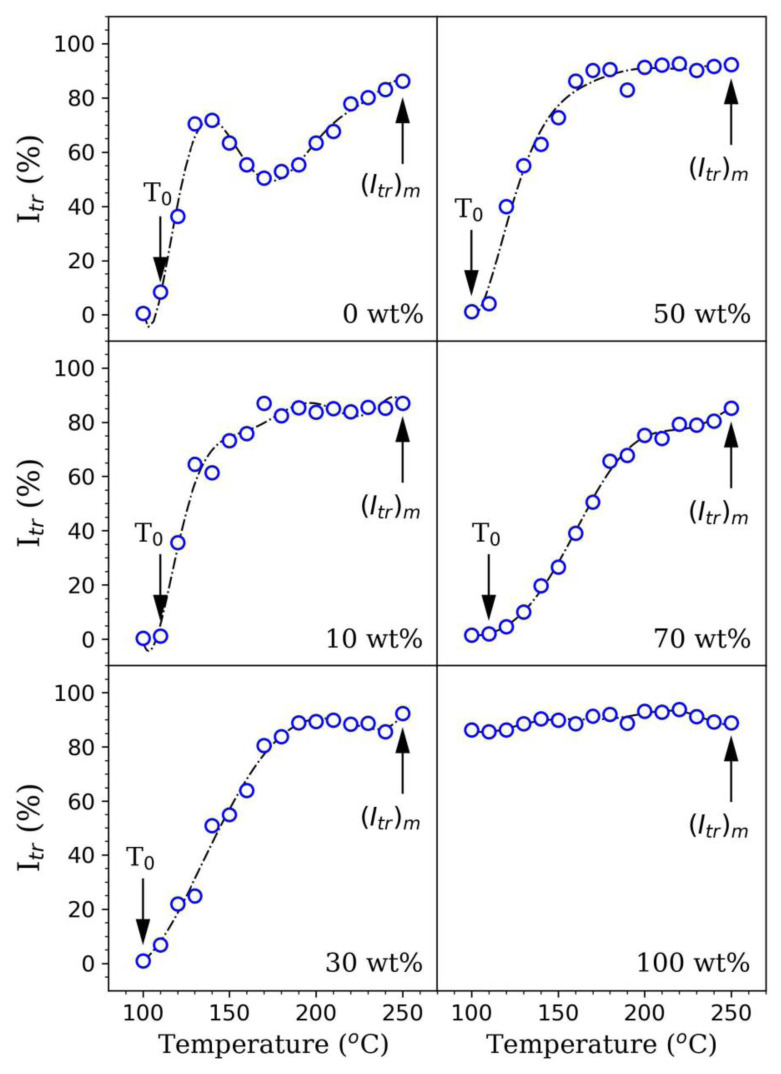
Transmitted light intensities, *I_tr_* at different annealing temperatures, *T* measured for PS latex/GO nanocomposite films containing different amounts of GO nanofillers.

Given Figure 3 illustrates the transmitted light intensities (*I_tr_*) measured at each annealing temperature for pure PS latex and PS latex/GO nanocomposite films, each having different mass fractions of GO nanosheets, respectively. Upon annealing process, *I_tr_* value begins to increase above the minimum film formation temperature, *T*_0_, and gets flatten for all film samples. On the other hand, for neat, GO-free (0 wt% of GO) PS latex film, *I_tr_* values first prone to increase for the specific annealing temperature varying from 100 °C to 130 °C, and then conversely decrease between 130 °C and 170 °C. Moreover, *I_tr_* values promote once more at the elevated annealing temperatures, particularly above 170 °C. However, *I_tr_* values of the films with GO content constantly enhance with an increase in annealing temperature. For each PS latex/GO nanocomposite film, the underlying mechanism behind such an increment in *I_tr_* above *T*_0_ is most likely the enhancement of the optical transparency of the latex films due to the sample annealing. All nanocomposite films are essentially composed of the high number of voids as well as the polymer-air boundaries before sample annealing, thereby, the incident light can be scattered from the polymer-air interface, which leads to decrease *I_tr_* values of the films.

The annealing process results in, first, the voids closure as a result of the viscous flow of latex particles. Afterward, it gives rise to the healing mechanism of particle-particle boundaries [22]. For this reason, as also seen from Figure 3, *I_tr_* values promote at higher annealing temperatures. Moreover, annealing the films particularly at the elevated temperatures results in healing and interdiffusion phenomenon, and these film formation mechanisms yield more transparent film structures. Besides, the fact that the enhancement of *I_tr_* above *T*_0_ basically refers to the void closure mechanism up to a certain annealing temperature value of *T_h_* at which the healing process usually takes place. Above *T_h_* point, why *I_tr_* values of PS latex/GO nanocomposite films promote can be elucidated well through the interdiffusion mechanism emerging between the polymer chains [23].

**Figure 4:**
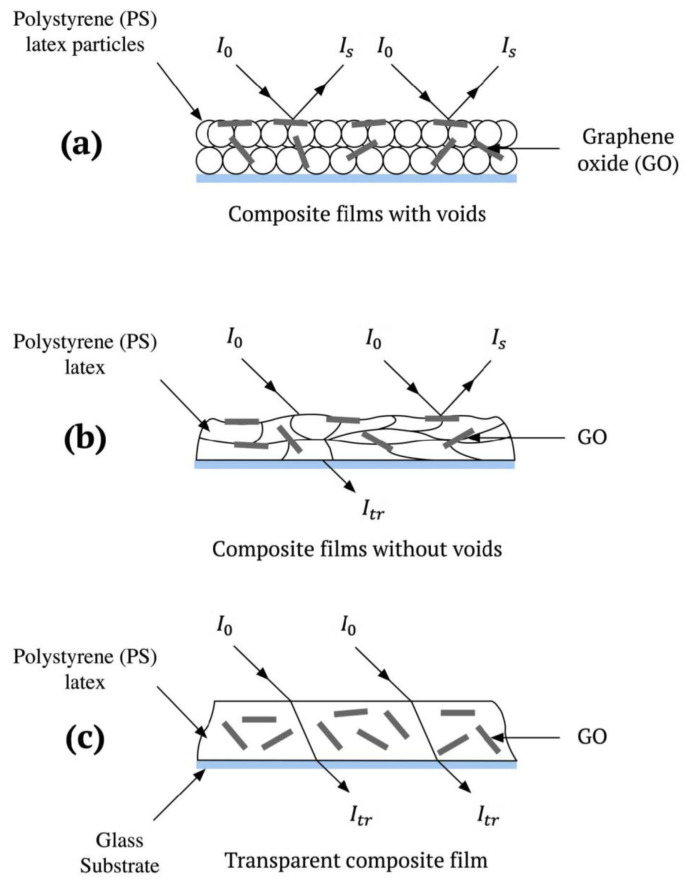
A cartoon representation to explain the film formation mechanisms: (a) composite films with voids, (b) composite films without voids, and (c) transparent film.

The behavior of *I_tr_* during the annealing process can be described with details as a schematic for PS latex/GO nanocomposite films in Figure 4. Here, Figure 4a represents the initial stage of the composite film formation. At this stage, the films in powder form and mainly contain high numbers of voids. The nanocomposite films, therefore, scatter the light, resulting in lower *I_tr_* values. Figure 4b illustrates the film structure where inter-particle voids disappear as a result of the annealing process. At this stage, *I_tr_* reaches its highest value. The reduction in *I_tr_* values for neat PS latex film at various annealing temperatures between 130 °C and 170 °C can be attributed to the light scattering phenomenon mainly arising from the PS latex particle aggregations formed in this film. Since the surface of PS latexes contains negative sulfate groups, steric repulsion, also called a repulsive Coulomb interaction, may emerge between these PS latex particles in the void closure stage at a certain distance. During the viscous flow, the overall effect of such a repulsive interaction can become more prominent and much stronger due to the majority of PS latex particles come closer, which may lead to the formation of PS latex aggregations.

**Figure 5:**
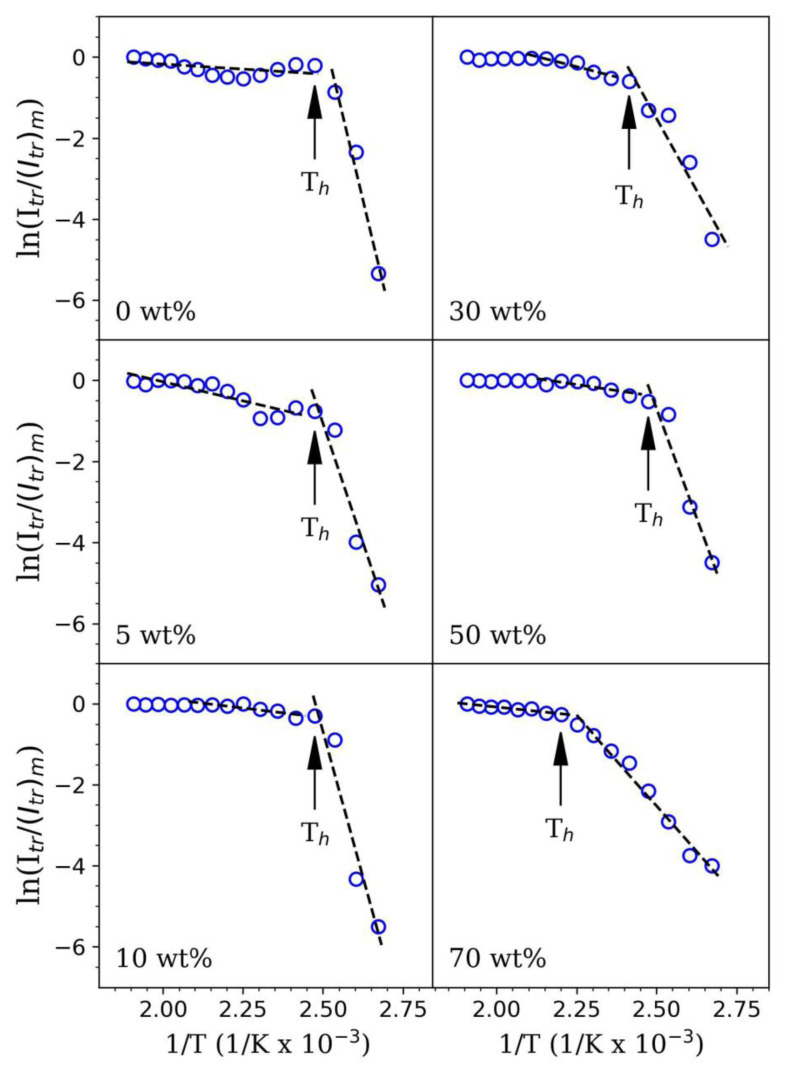
Measured *I_tr_*/(*I_tr_*)*_m_* values in log scale as a function of *T*^−1^ for the estimation of Δ*E* and Δ*H* energies.

In this respect, here, it can be said that the reduction in *I_tr_* values particularly above 130 °C for neat GO-free PS latex film is most likely due to light scattering from particle aggregations. Furthermore, the annealing process at much higher temperature values, particularly above 170 °C, leads to overcome such a steric repulsion phenomenon and all the voids in the film structure can be filled completely. Therefore, *I_tr_* values started to enhance once again above 170 °C. After the void-closure mechanism is done, the particle-particle interfaces are completely disappeared as a result of the interdiffusion of the polymer chains across the particle-particle interfaces. Thus, during the interdiffusion process, the nanocomposite coating turns into more transparent film and then *I_tr_* further increases once more at the elevated temperatures higher than that of 170 °C. In addition, one can also say that the amphiphilic features of GO can also lead to an increase in *I_tr_* values of GO-doped nanocomposite film samples. As GO contains both hydrophobic and hydrophilic (oxygen) functional groups on its molecular structure, GO nanolayers can easily absorb surface-active molecules [24]. One another possibility is that GO nanolayers may be attracted by PS latex particles as the Coulomb interaction can take place between the hydrophobic parts of GO and PS latex particles. For this reason, this physical phenomenon most probably hinder the repulsion between individual PS latex particles. The enhancement of *I_tr_* below and above *T_h_* value, as shown in Figure 5, can be further explained in detail by the voids closure and interdiffusion processes.

To better quantify the behavior of *I_tr_* between *T*_0_ and *T_h_*, one needs to take into account the activation energies required for the void closure phenomenon, Δ*H* [25]. A theoretical description of void closure model can be found from [26] with greater details. Using theoretical void closure model, *I_tr_* can be written as a function of Δ*H* given by Equation 1 as follows:


(1) $$I_{tr}(T)=A\cdot exp\;\left(-\frac{3\Delta H}{k_{B}T}\right)$$

where *A* is constant, *k_B_* is Boltzmann constant and *T* represents the annealing temperature in Kelvin. Taking the logarithm of above equation, one can obtain Equation 2, as given below, and calculate Δ*H* values using photon transmission data.


(2) $$\text{ln}(I_{tr}(T))=\text{ln}(A)-\frac{3\Delta H}{k_{B}T}$$

**Figure 6:**
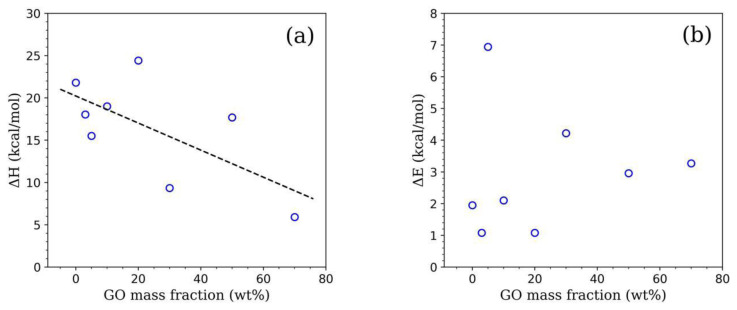
Changes in (a) viscous flow (Δ*H*) and (b) interdiffusion (Δ*E*) activation energies with respect to GO mass fraction (wt%). Black dashed straight line shown in (a) represents the linear fit applied to calculated Δ*H* data.

As theoretically described above, the enhancement in *I_tr_* values particularly above *T*0 arising mainly from the void closure mechanism. Therefore, for PS latex/GO nanocomposite films, above Equation 2 can be applied to *I_tr_* values measured at a specific temperature range between *T*_0_ and *T_h_*. As shown in Figure 5, the logarithmic form of *I_tr_* values were plotted as a function of *T*^−1^ for each film sample to be able to calculate Δ*H* (from the right-hand side) and all calculated Δ*H* values were given in Figure 6a and Table 2. The values shown in both Figure 6a and Table 2 have revealed that the Δ*H* values produced from linear fit curves prone to decrease from 21.80 kcal·mol^−1^ to 5.91 kcal·mol^−1^ upon the addition of GO nanofillers into PS matrices. This means that the amount of heat needed by only one mole of polymer to perform a jump in viscous flow is inversely proportional to the amount of GO nanofillers embedded within latex films. The decreasing trend of Δ*H* obtained for GO-added PS latex films agreed very well with Δ*H* values reported in other experimental studies. In another research, which similarly address the film formation and optical properties of PS/GO nanocomposites, Sunay et al. have also explored that Δ*H* values decreased from 0.86 kcal·mol^−1^ down to 0.42 kcal·mol^−1^ by increasing GO nanofiller concentration from 1 wt.% up to 25 wt.% within PS latex matrix [27]. The Δ*H* values reported in the present research were found 1–2 orders of magnitude higher than the reported values in their study. One of the possibilities for such magnitude difference in Δ*H* can be the latex particles employed in nanocomposite film design.

Since the PS latex particles used in [27] are labeled by fluorescent pyrene-(P) molecules, this may result in slightly smaller Δ*H* values compared to the obtained values reported in our research. Another underlying reason why higher Δ*H* values obtained in comparison with [27] can be the chemical composition of GO content used in PS latex nanocomposite films (as provided from different suppliers). Since GO nanolayers used in this research are 4–10% edge-oxidized, it is very likely that PS latex particles can adhere to the surface of GO nanolayers as a result of Coulomb interactions between latex particles and GO nanolayers. Wang et al. have reported that PS latex particles are prone to either attach on the edges of GO or can be covered by GO nanolayers [28]. This can indeed lead to increase the length of the voids between PS latex particles and much greater Δ*H* values are therefore required to take place the void closure process for our film samples.

Latex particle size is also another measure which can be taken into account to explain the difference in Δ*H* values. The larger the polymer particle size, the larger the voids between latexparticles will require much higher Δ*H* to fill the voids completely. In this study, the mean diameter of latex particles was measured as 540 nm, which is greater than that of the size of PS latex particles (324 nm) reported in [27]. This result also points out why higher Δ*H* values were obtained in our research.

A steady increment in *I_tr_* values particularly above *T_h_* was additionally described using the Prager–Tirrell (PT) model to further explain the interdiffusion mechanism of the polymer chains and a detailed context regarding the theoretical model can be found from [29] with details. According to the PT model, *I_tr_* can be expressed as a function of the activation energy of backbone motion, i.e. interdiffusion, Δ*E*, *k_B_* and *T* using the following Equation 3:


(3) $$I_{tr}(T)/I_{tr}(\infty )=A\cdot exp\;\left(-\frac{\Delta E}{2k_{B}T}\right)$$

Taking the logarithm of above Equation 3, one can apply the least-squares fitting method to the left-hand side of optical data presented in Figure 5 to produce Δ*E* values from the fit curves. Using this theoretical approach, Δ*E* was estimated for each nanocomposite film and they were listed in Table 2 and presented in Figure 6b as well. As one can see from both Figure 6b and Table 2 that the Δ*E* values varied between 1.08 kcal·mol^−1^ and 6.94 kcal·mol^−1^ without any trend and found not to change very much with the presence of GO nanolayers even at high concentrations. A similar random trend of the Δ*E* values was also observed in [27]. This result has indeed indicated that the amount of activation energy required for the mobility of polymer chains, i.e. interdiffusion of polymers, is independent of the amount of GO nanofillers within film composition. Polymer chain transfer process usually comes off at elevated annealing temperatures [30] and strongly dependent on the interactions between polymer and nanofiller content. At such a high temperature range, upon an increment in GO concentration within a polymeric medium, the added GO is more likely to hinder the latex particles to move freely as these colloidal particles are either bound to thesurfaces of GO nanolayers or coated by GO. After void closure process, since the latex films to a greater extend covered by GO nanolayers particularly, the chain transfer process becomes more easier to undergo complete interdiffusion with much less activation energy needed. Here, one can also notice that the calculated Δ*E* values are much smaller than that of Δ*H* valuesdue to a single polymer chain require much less activation energy in order to diffuse across the polymer-polymer interface compared to what bulk polymer needs. This also means that the film formation of GO-doped PS latex nanocomposite films most dominantly arouse from the void closure process.

On the other hand, *T*_0_ and *T_h_* values obtained from photon transmission measurements are also in a strong agreement with estimated Δ*H* and Δ*E*. Here, *T*_0_ is often known as the lowest temperature required for the onset of particle deformation leading to shrinking interstitial void diameters to sizes even smaller than the wavelength of light [31]. At annealing temperatures smaller than *T*_0_, the structure of PS latex film is dry, powdery, and not highly transparent. Yet, once the latex film sample is annealed at temperatures higher than *T*_0_, the film structure turns into homogeneous and optically more transparent [32]. For this reason, *T*_0_ can be considered as the critical temperature value where *I_tr_* values of the nanocomposite films often increase dramatically. On the other hand, for the particle-particle adhesion, the healing temperature, *T_h_*, is usually taken into account and *T_h_* can be easily estimated between two different linear regions (see Figure 5). By using the optical transmittance data as listed in Table 2 and presented in both Figure 3 and Figure 5, one can easily obtain *T*_0_ and *T_h_* values of nanocomposite films containing different GO content. From these experimental findings, it was clearly understood that *T_h_* values found for each nanocomposite film composition do not significantly change even in the presence of higher GO concentrations. This observation also pointed out that the interdiffusion mechanism of latex particles in the nanocomposite films that we fabricated is not dependent on the addition of GO nanolayers.

### 3.2. Morphological investigation of PS latex/GO nanocomposites

**Figure 7:**
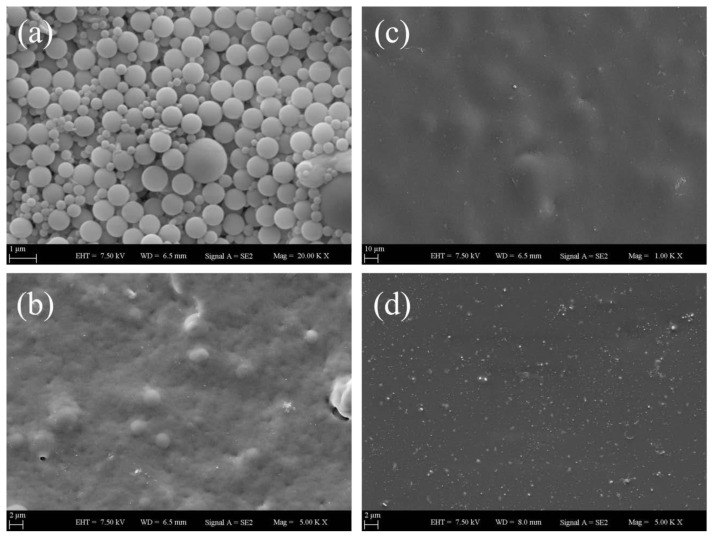
SEM images of pure (GO-free) PS latex film annealed at different temperatures: (a) 100 °C, (b) 130 °C, (c) 170 °C and (d) 250 °C.

Apart from photon transmission measurements performed to measure *I_tr_* values of nanocomposite films as well as to understand how optical transparency of nanocomposite film structures changes depending on the addition of GO nanolayers into latex particles, scanning electron microscopy (SEM) imaging method was additionally carried out to investigate how film morphology of the nanocomposites is affected in the presence of GO nanolayers at different annealing temperatures. The film morphology of pure, GO-free film made only from PS latex particles at various annealing temperatures is presented in Figure 7. Here, Figure 7a represents the SEM image of PS latex film annealed at 100 °C and high numbers of voids are clearly seen at this specific temperature. It is also observed that latex particle deformation is not seen due to annealing temperature is below *T_g_*, that is 105 °C. As shown in Figure 7b, upon annealing the same pure latex film at 130 °C, these voids have started to disappear along with the viscous flow and thereby *I_tr_* values increased. At a much higher annealing temperature of 170 °C as presented in Figure 7c, the void closure process of pure latex film has almost completed, however, *I_tr_* values have slightly decreased at this temperature. The underlying mechanism behind such a reduction in *I_tr_* can be explained well with undeformed, relatively large PS particles resulting in light scattering and these large latex particles can be seen from Figure 7c. Furthermore, Figure 7d represents the film morphology annealed at 250 °C and it is seen from SEM image that both void closure and interdiffusion processes have done completely; thereby, continuous, highly transparent, and quite smooth film surface couldbe obtained.

**Figure 8:**
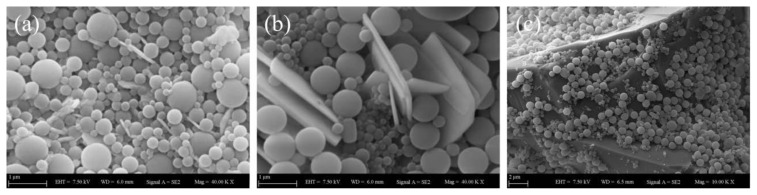
SEM images of PS latex/GO nanocomposite films annealed at 100°C, each containing (a) 10 wt%, (b) 30 wt%, and (c) 70 wt% of GO content.

**Figure 9:**
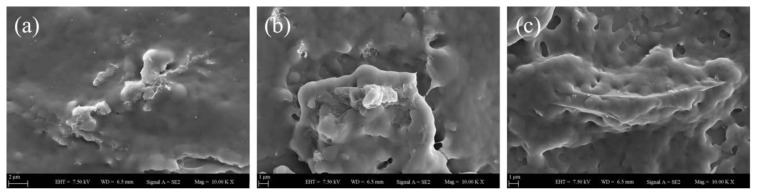
SEM images of PS latex/GO nanocomposite films annealed at 130°C, each containing (a) 10 wt%, (b) 30 wt%, and (c) 70 wt% of GO content.

**Figure 10:**
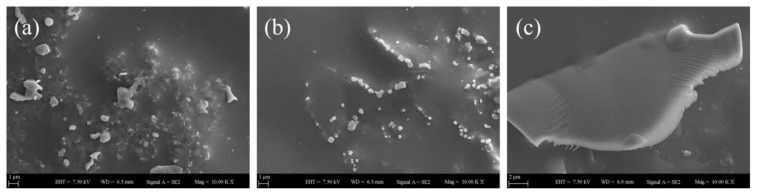
SEM images of PS latex/GO nanocomposite films annealed at 170°C, each containing (a) 10 wt%, (b) 30 wt%, and (c) 70 wt% of GO content.

**Figure 11:**
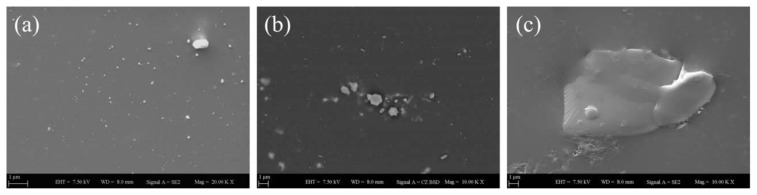
SEM images of PS latex/GO nanocomposite films annealed at 250°C, each containing (a) 10 wt%, (b) 30 wt%, and (c) 70 wt% of GO content.

Figures 8–11 represent the SEM images of GO-doped PS latex nanocomposite films annealed at specific temperatures of 100 °C, 130 °C, 170 °C and 250 °C, respectively where each film composed of 10 wt%, 30 wt% and 70 wt% of GO embedded within PS latex particles. As shown in Figure 8, each nanocomposite film annealed at 100 °C has random arrays of latex particles together with some porosity. Moreover, GO nanolayers are seen on the surface of latex particles for nanocomposite films which contain 10 wt% and 30 wt% of GO. As seen from Figure 8a and Figure 8b, they appeared as isolated sub-micrometer sticks or in forms of sheets embedded in latex particles. For 10 wt% and 30 wt% GO content film structures, one can also see that the GO nanolayers were homogeneously dispersed within PS latexes and no prominent aggregations have formed. Unlikely to this observation, as shown in Figure 8c, when 70 wt% of GO is added to PS latexes, the formation of a large GO block floating around latex particles can be seen on the film structure. It is most probably due to either the aggregation or the stacking of GO layers into relatively thicker flakes that may emerge during drying the film. Upon the addition of GO content up to 70 wt%, it is also possible thatGO nanolayers start to restock owing to strong interparticle interactions, the high aspect ratio and decreasing compatibility of GOwithin polymer matrices [33]. From these SEM images, one can observe that small fractions of latex particles were bound to the surface of GO nanolayers most likely due to Coulomb interactions taking place between latex particles and GO nanolayers. At this specific annealing temperature, that is 100 °C, the surface morphology of GO-doped nanocomposite films is remarkably affected upon the addition of nanofillers at higher concentrations.

Given Figure 9 shows the GO containing nanocomposite films annealed at 130 °C. At this temperature, it is seen that the latex particles have become more packed all over GO nanolayers making them compact film structures. These SEM images also support the experimental findings and clearly explain why measured *I_tr_* values promotedfor the nanocomposite films made up of GO nanofillers. When the annealing temperature was increased up to 170 °C, all GO containing film samples have become homogenous and had a smoother surface as shown in Figure 10. Particularly the films which contain 10 wt% and 30 wt% of GO fully covered by colloidal polymeric particles. Even though the surface of the films looks quite smooth and homogenous, both well-dispersed GO nanolayers and small fractions of GO blocks are still apparently seen on the film composition. Upon annealing the PS latex/GO films at the maximum temperature of 250 °C, as shown in Figure 11 that the film morphologies have become more continuous as well as flatter similar to a pure, GO-free latex film surface. The SEM images taken at such elevated annealing temperatures where all stages of latex film formation completely take place, pointed out that GO nanolayers were homogeneously dispersed and embedded within colloidal latex particles. For 70 wt% GO content at 250 °C (see Figure 11c), although a large piece of GO nanolayer embedded into continuous latex matrices was apparently seen on the surface of nanocomposite film, *I_tr_* value of nanocomposite film was measured above 80%. The SEM images together with photon transmission measurement results have demonstrated that our GO-doped PS latex films have excellent light transmission characteristics even at excessive GO nanofiller concentrations due to the presence of very uniform, well-dispersed GO nanolayers within PS matrices.

## 4. Concluding remarks

In this research, we used UVV and SEM techniques to study the effect of GO nanolayers on multi-staged film formation mechanisms and morphological properties of PS latex nanocomposite films. Nanocomposite blends and then the film samples, each containing different mass fractions of GO content ranging between 0 wt% and 70 wt%, were prepared using emulsion latex mixing and drop-casting methods, respectively. As the film formation mechanisms, void closure and Prager–Tirrel (interdiffusion) models were employed and characterized by using *I_tr_* data recorded for the film samples, each annealed at different temperatures varying from 100 °C to 250 °C. As a result, activation energy of viscous flow, Δ*H*, obtained from the photon transmission measurements decreased from 21.80 kcal·mol^−1^ down to 5.91 kcal·mol^−1^ through adding GO content up to 70 wt%, indicating that the required activation energy for void closure process is inversely proportional to GO concentration. However, activation energy of interdiffusion process, Δ*E*, randomly varied between 1.08 kcal·mol^−1^ and 6.94 kcal·mol^−1^ without any trend upon the addition of GO nanofillers into film composition, indicating that the added GO nanofillers had no significant effect on the backbone motion of latex polymer. Our theoretical findings were found to be quite consistentwith other studies reported in the literature. To keep track the film formation mechanisms and the dispersions of GO nanosheets and colloidal PS latex particles, SEM images were taken for different film compositions annealed at various temperatures. SEM images have demonstrated that well separated, homogeneously dispersed GO nanolayers within latex particles and very smooth film morphologies could be achieved even at high annealing temperatures. On the other hand, SEM images also revealed that PS latex particles were either adhered to the surfaces of GO orcoated by GO nanolayers, resulting in the restricted movement of latex particles and larger void distances between particles. In that sense, the SEM images were found to be in excellent agreement with calculated Δ*H* and Δ*E* values reported in this research paper. Even though the interdiffusion mechanism remained unaffected even at high mass fractions of GO, it was experimentally understood that thermally stable and highly transparent GO-doped PS latex nanocomposite films can be successfully obtained in the presence of GO nanofillers.

## Figures and Tables

**Table 1:** Average density and calculated thickness values of PS latex/GO nanocomposite films containing different GO nanofiller concentrations.

GO Content (wt%)	Average nanocomposite film density(g/ml)	Film thickness(*μm*)
0.0	1.050	6.72
3.0	1.054	7.11
5.0	1.059	7.27
10.0	1.076	7.38
20.0	1.093	7.48
30.0	1.13	67.53
50.0	1.179	7.69
70.0	1.222	7.72

**Table 2:** Activation energies of void closure (Δ*H*) and interdiffusion (Δ*E*) mechanisms, minimum film formation (*T*_0_)and healing temperature (*T_h_*) values obtained for PS latex/GO nanocomposite films made from various GO nanofiller concentrations.

GO Content (wt%)	Δ*H* (kcal·mol^−1^)	Δ*E* (kcal·mol^−1^)	*T*_0_ (°C)	*T_h_* (°C)
0.0	21.80	1.95	100	130
3.0	18.03	1.08	110	130
5.0	15.50	6.94	100	130
10.0	19.01	2.10	100	130
20.0	24.41	1.08	110	140
30.0	9.35	4.22	100	140
50.0	17.69	2.96	100	130
70.0	5.91	3.27	110	180
